# Optimizing Perpendicular Magnetic Anisotropy in MgO/CoFeB Structures Through Ultrathin CoFeB-Enhanced Ta Capping Layers

**DOI:** 10.1021/acsomega.4c11029

**Published:** 2025-05-01

**Authors:** Yu-Shen Yen, Chun-Liang Yang, Yung-Ling Chang, Chih-Huang Lai

**Affiliations:** 1 Ph.D. Program in Prospective Functional Materials Industry, National Tsing Hua University, Hsinchu 30013, Taiwan; 2 Department of Materials Science and Engineering, National Tsing Hua University, Hsinchu 30013, Taiwan

## Abstract

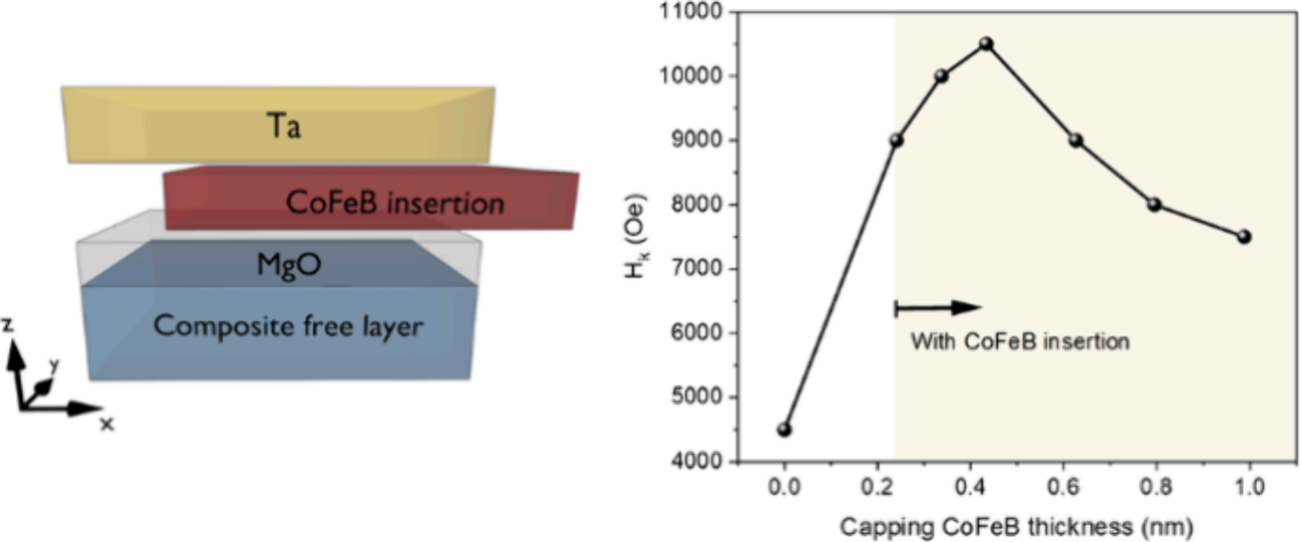

This study presents an innovative approach to optimizing perpendicular magnetic anisotropy (PMA) in CoFeB/MgO structures through the strategic insertion of an ultrathin CoFeB layer between the top capping layer (Ta or Mo) and the CoFeB/MgO stack. Adding a 0.43 nm CoFeB insertion layer significantly enhances PMA by improving Fe–O hybridization, suppressing interfacial diffusion, and stabilizing MgO crystallinity. Postannealing at 400 °C, the CoFeB (free)/MgO (capping)/CoFeB (0.43 nm insertion layer)/Ta (top capping) configuration demonstrates superior performance, achieving an interfacial anisotropy constant (*K_i_*) of 3.8 erg/cm^2^, the highest reported for similar structures under these conditions. Advanced analyses using high-resolution transmission electron microscopy and X-ray photoelectron spectroscopy reveal that the ultrathin CoFeB insertion effectively mitigates diffusion from the top capping layer, maintaining optimal oxidation and structural integrity at the interface. These findings not only deepen the understanding of PMA enhancement mechanisms but also provide a thermally stable, high-performance solution compatible with CMOS back-end-of-line processing. This work underscores the potential of interfacial engineering for advancing next-generation spintronic technologies.

## Introduction

Magnetic random-access memory (MRAM) has emerged as a promising solution for next-generation memory storage, offering nonvolatility, high speed, and scalability. Among various MRAM architectures, spin-transfer-torque magnetic random-access memory (STT-MRAM) with perpendicular magnetic anisotropy (PMA) is particularly promising due to its low power consumption and high thermal stability.^1,2^ The thermal stability of such devices is quantified by the thermal stability factor Δ = *E*_*b*_/*k*_*B*_*T*, where *E*_*b*_ is the energy barrier between two magnetization states, *k*_*B*_ is the Boltzmann constant, and *T* is the temperature. To retain data for 10 years for long-term storage, a thermal stability factor of 75 is required. Calculation shows a high interfacial anisotropy constant *K_i_* of 4.7 erg/cm^2^, which is required for 10 years, and 3.1 erg/cm^2^ for a 1 ms retention time, as device sizes scale down to 10 nm.^3^

CoFeB/MgO (capping) structures are foundational to PMA-STT-MRAM, with the top capping layer selection (Ta, Mo, or others) significantly affecting CoFeB PMA by influencing interfacial properties, including crystallinity, oxidation states, and magnetic dead layers. The first-principles calculation suggests that PMA arises from the hybridization between the 3d orbitals of Co and Fe and the 2p orbitals of oxygen in MgO^4^. A strong correlation between PMA and orbital hybridization of Fe 3d and O 2p was also experimentally observed with X-ray magnetic circular dichroism.^5^ The clean interface and optimal amount of oxygen at the interface are crucial to maintaining high PMA. Diffusion, oxidation, and structural integrity can disrupt this delicate interfacial balance. Much research has been conducted to engineer the CoFeB/MgO interface to enhance the PMA. Approaches such as reducing the top capping layer diffusion during annealing, minimizing sputtering damage to the capping layer, and controlling interfacial oxidation have been studied.^6−11^ For compatibility with CMOS back-end-of-line (BEOL) processing, MRAM stack structures must withstand annealing temperatures of up to 400 °C. Buffer and capping layers play crucial roles in stabilizing PMA under these conditions. Materials like Mo and W, with higher melting points and lower diffusivity, have been shown to preserve the CoFeB/MgO interface integrity during high-temperature annealing.^12−14^ These layers also act as boron sinks during annealing, enabling coherent tunneling between CoFe(001) and MgO(001) for high tunneling magnetoresistance (TMR).^15−18^ Simultaneously, reduced boron concentration at the interface promotes Fe–O and Co–O hybridization, which enhances the PMA.

Beyond acting as boron sinks, buffer and capping layers influence interfacial anisotropy, surface roughness, and texture, all of which contribute to higher PMA and TMR.^9,19−22^ The capping layer prevents oxidation during processing, but improper selection can lead to overoxidation, thereby degrading PMA.^23,24^ Materials like Mo and W are proposed to replace Ta as they exhibit greater annealing tolerance and reduced diffusion.^11−14,25^ The literature also reports that Ru and CoFeB capping layers exhibit better *K_i_* compared to Ta capping layers in the MgO/CoFeB/spacer/CoFeB/MgO/capping structure, which is attributed to better MgO layer integrity after annealing.^26,27^ With increasing Ta capping thickness, the top MgO layer appears thinner under TEM contrast, despite having the same nominal thickness. This indicates degradation of the top MgO layer due to Ta diffusion and MgO reduction. Additionally, the use of ultrathin insertion layers has been explored to suppress diffusion and mitigate sputtering damage from the capping layer, further improving PMA.^6,28^

Enhancing the PMA of CoFeB (free)/MgO systems requires preserving interfacial integrity, optimizing Fe–O and Co–O hybridization, suppressing diffusion during annealing, and precisely controlling the oxygen profile during deposition. This study introduces a novel strategy that addresses these challenges by incorporating an ultrathin CoFeB layer between the capping layer (Ta or Mo) and the CoFeB (free)/MgO stack. This innovative approach not only modifies the oxidation profile but also effectively suppresses interfacial diffusion and enhances Fe–O hybridization. Additionally, it minimizes excess oxygen at the interface and preserves capping MgO crystallinity, significantly improving PMA. Among the tested configurations, the MgO (capping)/ultrathin CoFeB/Ta (top capping) structure emerges as the most effective, achieving superior PMA compared to its Mo-based counterpart. These findings not only deepen our understanding of PMA enhancement mechanisms but also highlight the critical importance of interfacial engineering. Furthermore, they provide a practical pathway for designing thermally stable, high-performance spintronic devices that are fully compatible with CMOS back-end-of-line processing, advancing the development of next-generation spintronic technologies.

## Experimental Section

**Figure 1:**
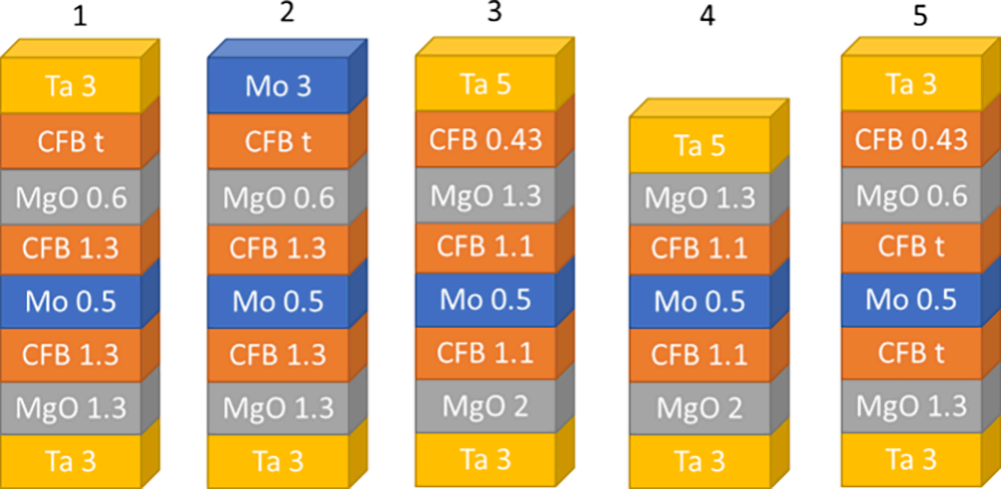
Schematic diagram of Stacks 1 to 5. Numbers indicate nominal thicknesses in nanometers.

All samples were prepared on Si substrates with a 200 nm thermal oxide layer using a magnetron sputtering system with a base pressure of 2 × 10^–8^ Torr. MgO was sputtered using RF sputtering, while other materials were deposited using DC sputtering. The composite free layer structure consisted of CoFeB/Mo/CoFeB. An MgO capping layer was added on top of the top CoFeB layer to enhance the PMA of the top CoFeB layer. The composition of CoFeB is Co_20_Fe_60_B_20_ in atomic percent. The stack structures are shown in Figure 1, and the functions for each layer are shown in stack 1.

Stack 1:Si/SiO_2_/Ta (adhesion layer 3)/MgO (barrier layer 1.3)/CoFeB (free 1.3)/Mo (0.5)/CoFeB (free 1.3)/MgO (capping layer 0.6)/CoFeB (ultrathin insertion layer t)/Ta (top capping 3).

Stack 2: Si/SiO_2_/Ta (3)/MgO (1.3)/CoFeB (1.3)/Mo (0.5)/CoFeB (1.3)/MgO (0.6)/CoFeB (t)/Mo (3).

Stack 3: Si/SiO_2_/Ta (3)/MgO (2)/CoFeB (1.1)/Mo (0.5)/CoFeB (1.1)/MgO (1.3)/CoFeB (0.43)/Ta (5).

Stack 4: Si/SiO_2_/Ta (3)/MgO (2)/CoFeB (1.1)/Mo (0.5)/CoFeB (1.1)/MgO (1.3)/Ta (5).

Stack 5: Si/SiO_2_/Ta (3)/MgO (2)/CoFeB (0.5t)/Mo (0.5)/CoFeB (0.5t)/MgO (0.6)/CoFeB (0.43)/Ta (3).

The numbers in parentheses are nominal thicknesses in nanometers. The film thickness was calibrated by using an atomic force microscope (AFM). The samples were annealed in a vacuum furnace at a base pressure of 2 × 10^–5^ Torr at 400 °C for 1 h to promote the crystallization of CoFeB to achieve PMA and meet the CMOS BEOL thermal budget requirements. Magnetic properties were measured by using a vibrating sample magnetometer (VSM). The chemical state analysis and elemental depth profiling were carried out by using X-ray photoelectron spectroscopy (XPS). High-resolution transmission electron microscopy (TEM) was used to evaluate the crystallinity and thickness of the MgO and CoFeB layers, both with and without ultrathin CoFeB insertion. TEM imaging provided detailed insights into the structural differences that the Ta and Mo capping layers imparted.

## Results and Discussion

### Enhanced PMA in CoFeB/Ta Capped Structures

**Figure 2:**
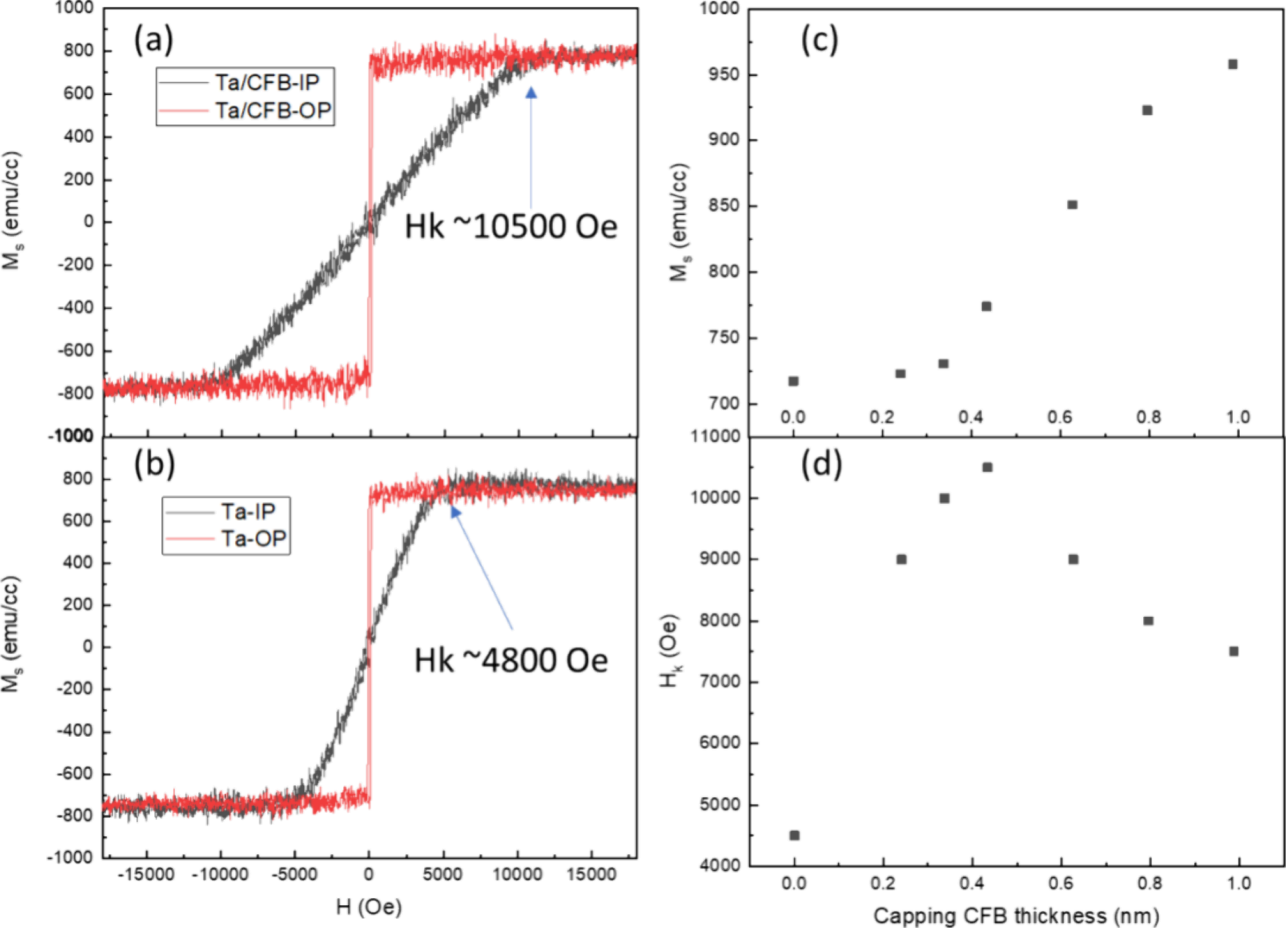
Out-of-plane and in-plane hysteresis loops of the stack 1 sample: (a) with a capping structure of MgO/CoFeB (0.43 nm insertion)/Ta and (b) with a capping structure of MgO/Ta. The dependence of the inserted CoFeB (CFB) thickness on (c) *M_s_* and (d) *H_k_* in the stack 1 sample.

Based on the previous work where Fe insertion reduced interdiffusion from the top capping W layer,^6^ we examined CoFeB insertion before Ta capping to assess its impact on PMA. The hysteresis loops of stack 1 with a CoFeB insertion thickness of 0.43 and 0 nm (no CoFeB insertion) are shown in Figure 2a,b. Inserting CoFeB before Ta deposition results in a significantly higher saturation field *H_k_*. To compare the perpendicular anisotropy of both structures, effective magnetic anisotropy energy density *K*_eff_ was calculated by *K*_eff_ = *M*_*s*_ × *H*_*k*_/2, where M_s_ is saturation magnetization, and *H_k_* is obtained from the saturation field of the hard-axis hysteresis loop. The *K*_eff_ for the structure with 0.43 nm CoFeB insertion is 4.06 Merg/cm^3^, while that of the structure without CoFeB insertion is 1.75 Merg/cm^3^. The M_s_ did not increase until the capping CoFeB thickness exceeded 0.33 nm, as shown in Figure 2c,d, while the *H_k_* increased dramatically as we inserted CoFeB before Ta capping. The ultrathin CoFeB insertion at low thickness likely forms a magnetic dead layer, which explains why M_s_ does not increase with the CoFeB insertion. The calculation of *M_s_*, *K*_eff_, and *K_i_* did not include the thickness of the capping CoFeB. Boron diffusion in CoFeB during the annealing process significantly influences changes in M_s_. Since the change in M_s_ with ultrathin CoFeB insertion is small, we believe that boron diffusion is similar across our series of samples. The highest *K*_eff_ is observed around a CoFeB capping thickness of 0.43 nm. This is presumably due to the optimal thickness balancing diffusion blocking from the top Ta layer during annealing with the development of in-plane anisotropy of the capping CoFeB. The challenge of ultrathin CoFeB to form layer continuity and the interdiffusion between the top capping layer and CoFeB after high-temperature annealing may lead to in-plane anisotropy (IMA) at very thin CoFeB thickness, even when it exceeds the dead layer thickness.^7^ Thus, the gradual degradation of *K*_eff_ with increasing CoFeB insertion thickness larger than 0.43 nm is attributed to the initial development of in-plane anisotropy in the capping CoFeB.

**Figure 3:**
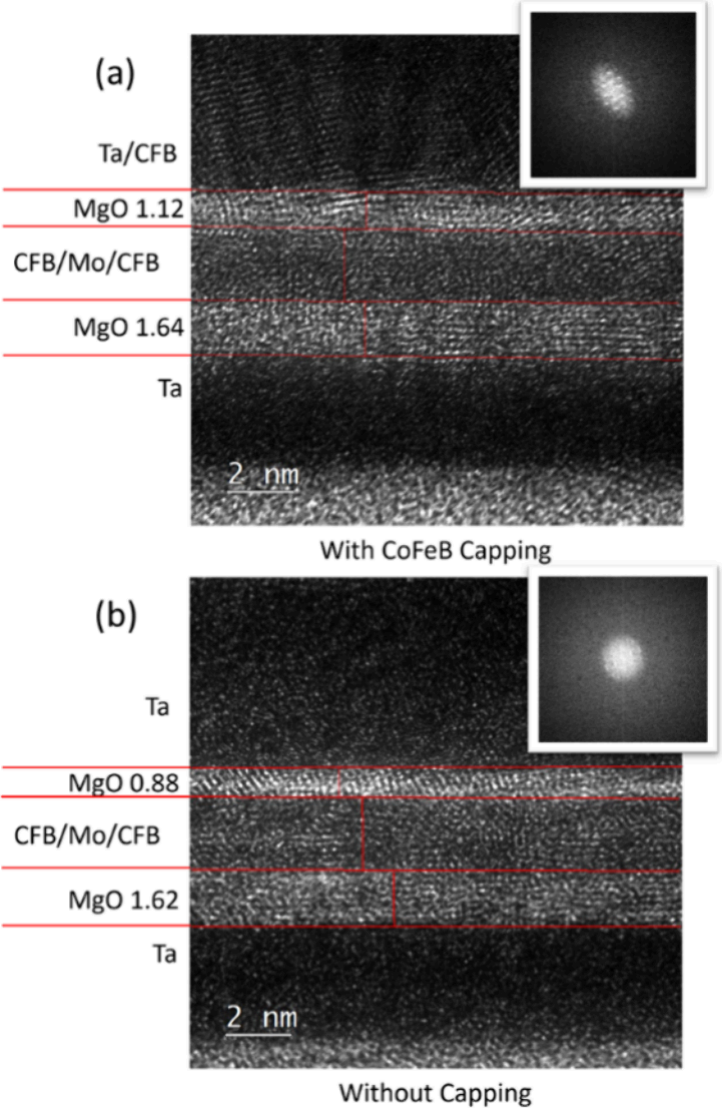
High-resolution TEM images of (a) stack 3 sample with CoFeB insertion and (b) stack 4 sample without CoFeB insertion. The insets show FFT of the top Ta capping layer image.

Stacks 3 and 4 were prepared for TEM analysis to evaluate crystallinity and diffusion behavior. It is very challenging to analyze the thickness variations of very thin 0.6 nm MgO due to the diffused interface; therefore, the capping MgO thickness in these two stacks is increased from 0.6 to 1.3 nm for enhanced TEM contrast. We also compare *K*_eff_ in structures with a 1.3 nm top MgO layer capped with Ta and CoFeB/Ta. While the results also show an increase in *K*_eff_ with CoFeB insertion, both structures exhibit lower *K*_eff_ compared to the 0.6 nm top MgO structure. A previous study on RF-sputtered MgO and DC-sputtered Mg with oxygen flow shows that the latter method results in as-deposited PMA, whereas RF-sputtered MgO exhibits as-deposited IMA due to overoxidation.^29^ The lower *K*_eff_ may be due to excess oxygen in the thicker RF-sputtered MgO layer.^23^ Besides the insertion of the CoFeB capping layer, the structures of the two stacks are identical, and both were annealed at 400 °C for 1 h. TEM analysis in Figure 3 shows that samples with CoFeB insertion retain a thicker MgO capping layer. The stack with CoFeB insertion has a capping MgO thickness of 1.12 nm, and the stack without insertion has a thickness of 0.88 nm, while the bottom MgO barrier layer thickness is quite similar (1.62 nm vs 1.64 nm) between the two stacks. The thickness difference in the top capping MgO may come from variations in Ta diffusion. Previous literature also shows a thicker MgO thickness contrast under TEM with suppressed diffusion or intermixing from the top layer.^6,27^ The CoFeB insertion layer may effectively suppress the diffusion of Ta into the CoFeB (free)/MgO interface. Additionally, the Ta capping layer appears to have improved crystallinity, which may also contribute to reduced diffusion from the top Ta. The insert at the top-right corner of Figure 3 shows the fast Fourier transform (FFT) of the top Ta layer image. The one with CoFeB insertion shows some diffraction spots, while the one without is more diffuse. The reduced Ta diffusion into the CoFeB (free)/MgO interface by the CoFeB insertion leads to higher PMA.

### Oxidation States and Their Impact on PMA

**Figure 4:**
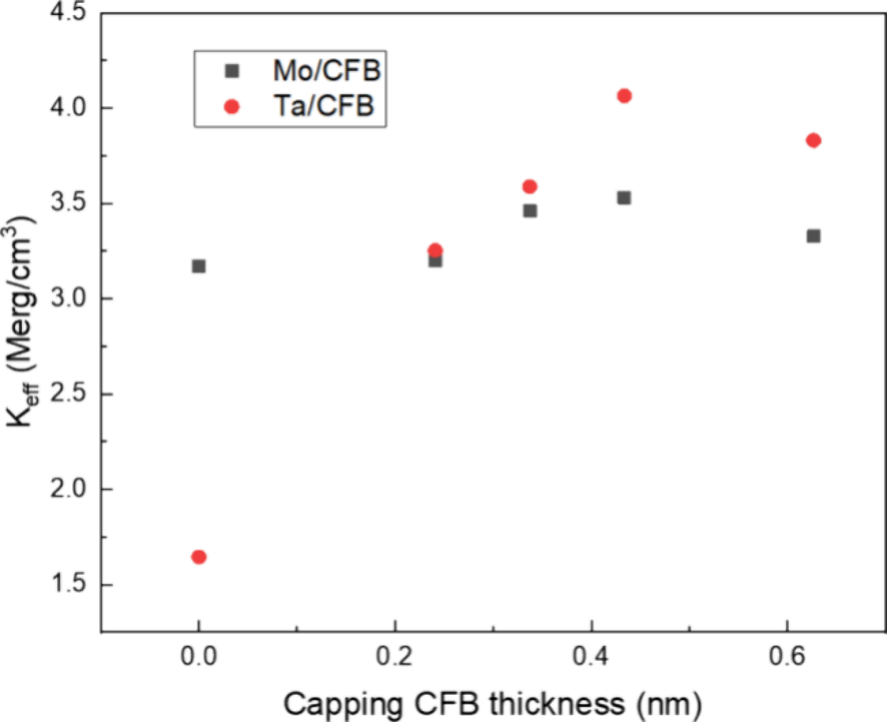
Variations of *K*_eff_ with the capping CoFeB thickness for CoFeB/Ta capping (stack 1) and CoFeB/Mo capping (stack 2).

To enhance PMA with an annealing temperature above 350 °C, previous studies have suggested that W and Mo as buffer/capping layers are more stable than Ta.^13,14,30^ We thus further investigate the two stack structures composed of the Ta (Stack 1) and Mo (Stack 2) capping layers with varying CoFeB capping layer thicknesses. The *K*_eff_ of both series of samples is shown in Figure 4. Without CoFeB insertion, the Mo capping layer exhibits a higher *K*_eff_ than the Ta capping layer, which may result from less diffusion of Mo during the high-temperature annealing.^14^ On the other hand, with the CoFeB insertion before Ta and Mo deposition, the *K*_eff_ variations are less pronounced, but CoFeB/Ta combined capping exhibits higher anisotropy than the CoFeB/Mo combined capping.

**Figure 5:**
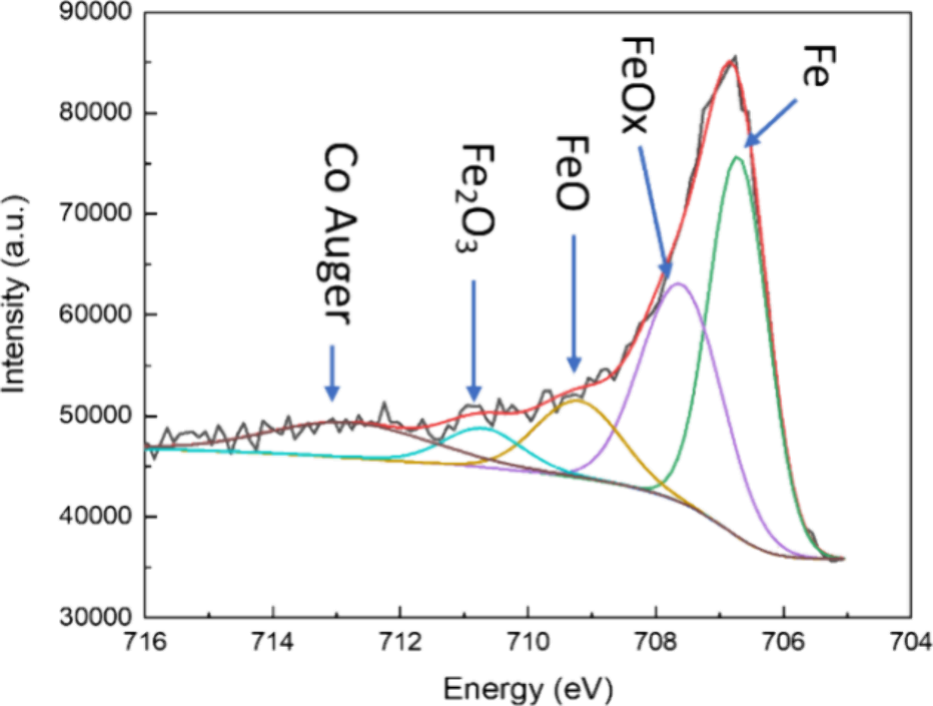
XPS of Fe 2p_3/2_ for the Si/SiO_2_/Ta (3)/MgO (1.3)/CoFeB (free 1.3)/Mo (0.5)/CoFeB (free 1.3)/MgO (0.6)/CoFeB (0.43)/Ta (3) structure. The peaks correspond to Fe, FeO_*x*_, FeO, Fe_2_O_3_, and the Co Auger peak. The spectrum was taken at the interface between the capping MgO and the top composite free layer.

The enhanced *K*_eff_ for the CoFeB/Ta capping layer compared with that of the CoFeB/Mo capping layer cannot be solely explained by the reduction of diffusion from Ta (or Mo) because Mo should have less diffusion than Ta. To further analyze the differences between the CoFeB/Ta and CoFeB/Mo capping structures, we performed XPS analysis on both samples, as well as a reference sample with Ta capping but no CoFeB insertion. Figure 5 presents the representative high-resolution XPS spectrum of the Fe 2p_3/2_ peak of the MgO/CoFeB/Ta capping structure and its fitting curve. A series of high-resolution XPS curves is obtained with Ar milling on the sample surface to conduct depth profiling. The data shown in Figure 5 correspond to the position at the top composite free layer CoFeB and MgO interface, providing a better representation of the interfacial anisotropy. The peak positions at 706.7, 707.6, 709.2, 710.7, and 712.9 eV correspond to Fe, FeO_*x*_ (*x* < 1), FeO, Fe_2_O_3_, and the Co Auger peak according to the XPS handbook.^31^ We can calculate the integrated area ratio of all Fe oxides to Fe (*R* = S_Fe–O_/S_Fe_) with the fitting peaks to determine the relative oxide content. The magnetic properties and oxidation ratio *R* of the three stacks are shown in Table 1.

**Table 1:** Comparison of Oxidation Ratio *R* and *K*_eff_ for Different Capping Structures

capping structure	*H_k_* (Oe)	*K*_eff_ (erg/cm^3^)	*R*
CoFeB/Ta	10500	4.06	1.24
Ta	4800	1.75	1.05
CoFeB/Mo	7000	2.97	1.43

According to the first-principles calculation, over- or under-oxidation can lead to suboptimal PMA at the Fe–O interface.^4^ The R of CoFeB/Mo is 15% higher than that of the CoFeB/Ta capping stack, and the Ta-only capping is 18% lower compared to the CoFeB/Ta capping. Based on the *K*_eff_ and R values for the three stacks, we suggest that the CoFeB/Mo capping may be overoxidized, while the Ta capping is under-oxidized. The CoFeB/Ta capping demonstrates the highest *K*_eff_ with an intermediate oxidation ratio.

**Figure 6:**
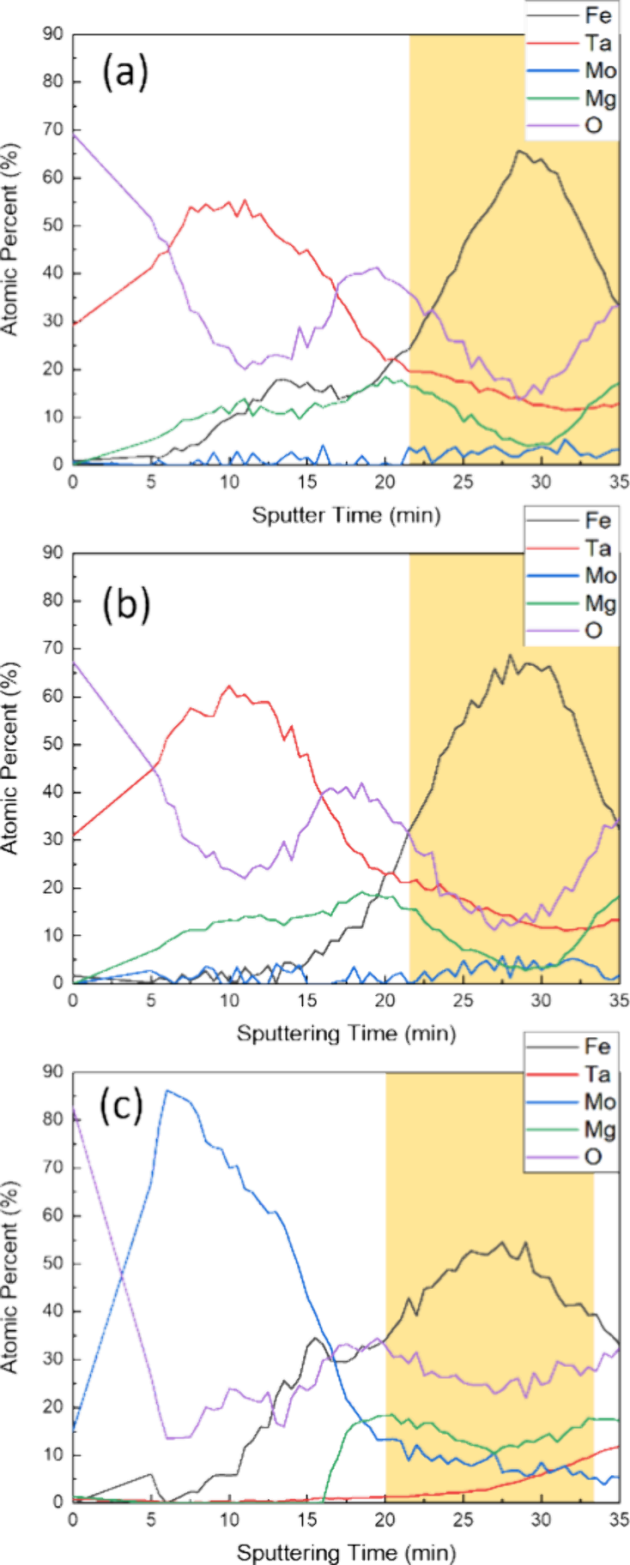
XPS depth profiles of (a) CoFeB/Ta capping, (b) Ta capping, and (c) CoFeB/Mo capping. The highlighted region represents the composite free layer position.

Depth profiling results of the three stacks are shown in Figure 6. The calculation of the atomic concentration is based on the elemental peak intensity of each element, with sensitivity factor correction. The Ta concentration at the top CoFeB (free)/MgO interface is approximately 19.5% for the CoFeB/Ta capping structure and around 21% for the Ta capping structure, which also aligns with previous TEM results, indicating reduced diffusion. In contrast, the CoFeB/Mo capping shows a sharp Mo termination at the interface, consistent with literature findings that Mo exhibits less diffusion compared to Ta. The highlighted region marks the composite free layer position, indicating that oxygen levels were highest in CoFeB/Mo, moderate in CoFeB/Ta, and lowest in Ta-capped samples. This is consistent with the XPS Fe 2p_3/2_ findings. The standard enthalpies of formation for Ta_2_O_5_, MoO_2_, MoO_3_, Fe_3_O_4_, Co_2_O_3_, and MgO are −2045.98, −587.85, −703.73, −1118.4, −577, and −602 kJ/mol, respectively. Ta likely formed tantalum oxide, and thus it can be a good oxygen sink to reduce excess oxygen at the CoFeB (top composite free layer)/MgO (capping) interface. Previous research suggests that both tantalum and molybdenum are highly refractory materials. The large enthalpy of formation difference between tantalum oxide and MgO leads to interdiffusion. Molybdenum oxides, on the other hand, have a much more similar enthalpy of formation compared to MgO, resulting in negligible interdiffusion.^14^ Without the CoFeB insertion, Ta may diffuse in and absorb too much oxygen, reducing the Fe–O hybridization between the capping MgO and the top composite CoFeB free layer. With the insertion of ultrathin CoFeB, the Ta diffusion is suppressed and the amount of oxygen at CoFeB (free)/MgO is optimized for the Fe–O hybridization. On the other hand, the less negative standard formation enthalpy of molybdenum oxide results in higher oxygen content at the CoFeB (free)/MgO interface and in the composite free layer. The elevated oxygen content in CoFeB/Mo likely contributes to weaker PMA than CoFeB/Ta, as overoxidation results in reduced PMA. It has been reported that a thick MgO capping layer can lead to overoxidation, thereby diminishing the PMA at the CoFeB (free)/MgO interface.^23^ The choice of CoFeB/Ta or CoFeB/Mo would largely depend on the stack oxidation condition. This opens up a new adjustable parameter for PMA with existing STT-MRAM stack materials.

**Figure 7:**
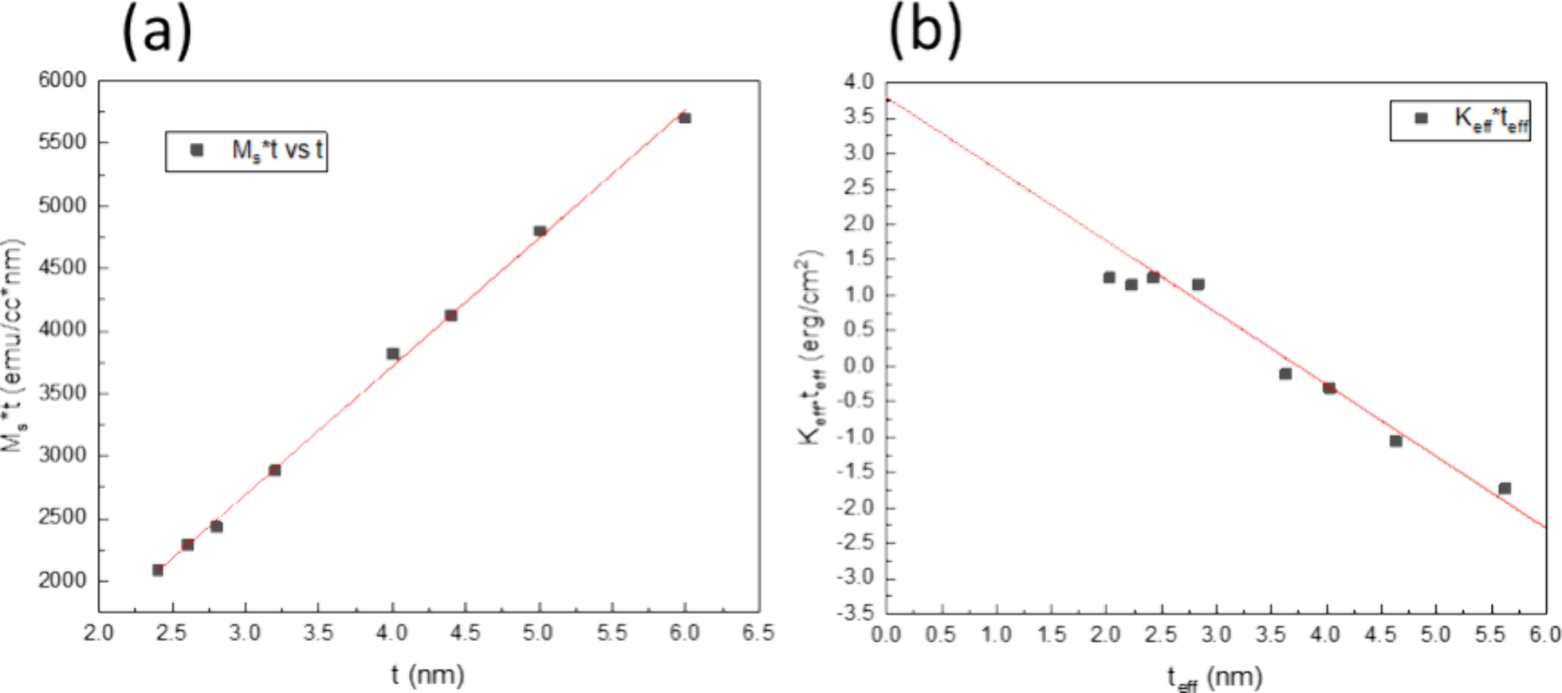
(a) *M_s_* × *t* vs *t* and (b) *K*_eff_ × *t*_eff_ vs *t*_eff_ with varied composite CoFeB free layer thickness *t* (CoFeB 0.5t/Mo/CoFeB 0.5t). A high *K_i_* of 3.8 erg/cm^2^ is achieved with CoFeB capping insertion.

To extract the interfacial anisotropy energy in the best PMA samples with a CoFeB/Ta capping, we varied the CoFeB thickness of the composite free layer (Stack 5 samples). We chose a capping structure of CoFeB (0.43)/ Ta (3) as it revealed the highest *K*_eff_ and varied composite CoFeB thickness. The upper and lower CoFeB layers in the composite free layer are assumed to have the same M_s_ and dead layer thickness. Figure 7a shows *M_s_* × *t* vs *t*. *t* is the total nominal CoFeB composite free layer thickness. From the linear fitting, we can obtain the dead layer thickness (*t*_dead_) and *M_s_* of CoFeB. The effective magnetic layer thickness is defined as *t*_eff_ = *t* – *t*_dead_. *K_i_* is determined using linear extrapolation of the linear region (*t*_eff_ ≥ 2.83 nm) in Figure 7b, following the formula: , where *K_b_* is the bulk magnetic anisotropy. The intercept of the linear extrapolation represents the *K_i_* value of the system. Fitting of Figure 7a shows a dead layer thickness of 0.37 nm and an *M_s_* value of 1025 emu/cc. A very large *K_i_* of 3.8 erg/cm^2^ is achieved based on the fitting of the linear region of Figure 7b. The highest *K_i_* for a single CoFeB/MgO interface reported to date is 2.05 ± 0.07 erg/cm^2^ with the Mo/Co_40_Fe_40_B_20_/MgO structure^14,32^ and the highest *K_i_* for double CoFeB/MgO interfaces with the structure of Ta/MgO/Co_20_Fe_60_B_20_/Mo/Co_20_Fe_60_B_20_/MgO/Ta reported to date is 3.2 erg/cm^2^, annealed after 400 °C.^25^

## Conclusions

This study establishes the significant advantage of inserting an ultrathin CoFeB layer before Ta capping in enhancing perpendicular magnetic anisotropy (PMA) in composite free-layer structures. Among the tested configurations, the CoFeB/Ta combination demonstrated exceptional performance, achieving a high effective anisotropy energy density (*K*_eff_) of 4.06 Merg/cm^3^ and an interfacial anisotropy constant (*K_i_*) of 3.8 erg/cm^2^, the highest reported for similar structures annealed at 400 °C. These values not only meet but exceed the requirements for CMOS back-end-of-line (BEOL) processing, positioning this configuration as a breakthrough for next-generation spintronic devices. The enhanced PMA in the CoFeB/Ta capping system is attributed to a synergy of critical factors: optimized oxidation at the CoFeB (top composite free layer)/MgO (capping) interface and reduced Ta diffusion, which preserves interfacial and structural integrity. While CoFeB/Mo also showed promise, its tendency toward overoxidation limited its performance compared to the CoFeB/Ta-based configuration. Moreover, this study identified 0.43 nm as the optimal CoFeB insertion thickness, which effectively blocks diffusion, while mitigating the development of in-plane anisotropy. This careful thickness optimization further underscores the importance of precise interfacial engineering in achieving thermally stable and high-performance magnetic properties.

These findings highlight the transformative potential of the MgO/CoFeB/Ta configuration as a robust and scalable solution for spintronic applications. Its compatibility with high-temperature BEOL processing and superior magnetic properties make it a strong candidate for integration into next-generation MRAM devices, paving the way for advancements in memory technology and broader spintronic innovations.

## Supporting Information Available

Hysteresis loop (CoFeB 0.43 nm/Mo); high-resolution XPS spectra (CFB/Mo and Ta) (PDF)
