# Supplementary material for: Optimizing Perpendicular Magnetic Anisotropy in MgO/CoFeB Structures Through Ultrathin CoFeB-Enhanced Ta Capping Layers
Source: ACS Omega. 2025 May 1;10(18):18510–6. doi: 10.1021/acsomega.4c11029 (PMC12079193; doi:10.1021/acsomega.4c11029)
Supplement: Supplementary file 1 — ao4c11029_si_001.pdf [file ao4c11029_si_001.pdf]

Supporting Information

Optimizing Perpendicular Magnetic Anisotropy in MgO/CoFeB Structures Through Ultra-thin CoFeB-Enhanced Ta Capping Layers

Yu-Shen Yen[1,2], Chun-Liang Yang[2], Yung-Ling Chang[2], Chih-Huang Lai[2]*

[1]Ph.D. Program in Prospective Functional Materials Industry, National Tsing Hua University, Hsinchu 30013, Taiwan
[2]Department of Materials Science and Engineering, National Tsing Hua University, Hsinchu 30013, Taiwan

CONTAINS

Figure S1. Hysteresis loop of CoFeB (0.43 nm)/Mo capping structure
Figure S2. High resolution XPS spectrum of (a) CFB/Mo and (b) Ta capping structure

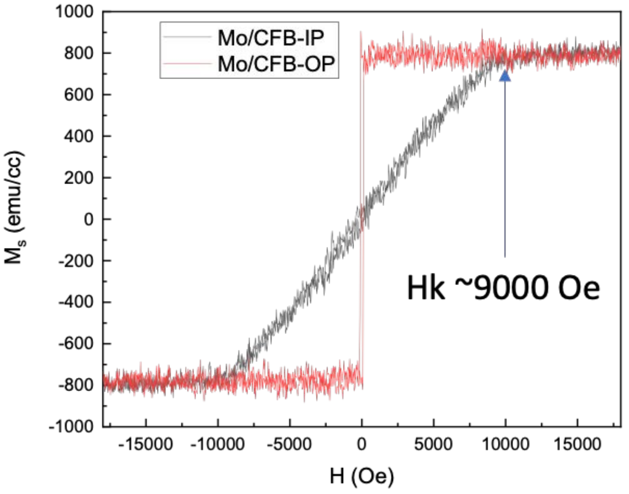

Figure S1. In-plane and out-of-plane hysteresis loop of CoFeB/Mo capping structure.

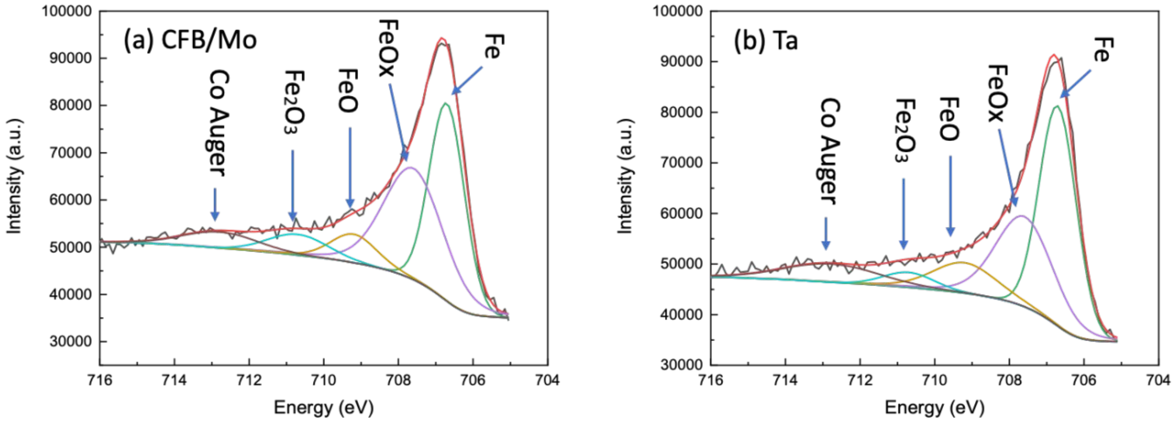

Figure S2. Fe 2p 3/2 XPS spectrum of (a) CFB/Mo and (b) Ta capping structure.
